# Deep Learning-Based Automatic Assessment of Lung Impairment in COVID-19 Pneumonia: Predicting Markers of Hypoxia With Computer Vision

**DOI:** 10.3389/fmed.2022.882190

**Published:** 2022-07-26

**Authors:** Yauhen Statsenko, Tetiana Habuza, Tatsiana Talako, Mikalai Pazniak, Elena Likhorad, Aleh Pazniak, Pavel Beliakouski, Juri G. Gelovani, Klaus Neidl-Van Gorkom, Taleb M. Almansoori, Fatmah Al Zahmi, Dana Sharif Qandil, Nazar Zaki, Sanaa Elyassami, Anna Ponomareva, Tom Loney, Nerissa Naidoo, Guido Hein Huib Mannaerts, Jamal Al Koteesh, Milos R. Ljubisavljevic, Karuna M. Das

**Affiliations:** ^1^Department of Radiology, College of Medicine and Health Sciences, United Arab Emirates University, Al Ain, United Arab Emirates; ^2^Abu Dhabi Precision Medicine Virtual Research Institute (AD PM VRI), United Arab Emirates University, Al Ain, United Arab Emirates; ^3^Department of Computer Science and Software Engineering, College of Information Technology, United Arab Emirates University, Al Ain, United Arab Emirates; ^4^Big Data Analytics Center, United Arab Emirates University, Al Ain, United Arab Emirates; ^5^Eye Microsurgery Center “Voka”, Minsk, Belarus; ^6^Biomedical Engineering Department, College of Engineering, Wayne State University, Detroit, MI, United States; ^7^Siriraj Hospital, Mahidol University, Nakhon Pathom, Thailand; ^8^Department of Neurology, Mediclinic Parkview Hospital, Dubai, United Arab Emirates; ^9^Department of Clinical Science, College of Medicine, Mohammed Bin Rashid University of Medicine and Health Sciences, Dubai, United Arab Emirates; ^10^College of Medical Sciences, Ras Al Khaimah Medical Health and Sciences University, Ras Al Khaimah, United Arab Emirates; ^11^Department of Computer Science, Abu Dhabi Polytechnic, Abu Dhabi, United Arab Emirates; ^12^Scientific-Research Institute of Medicine and Dentistry, Moscow State University of Medicine and Dentistry, Moscow, Russia; ^13^Department of Public Health and Epidemiology, College of Medicine, Mohammed Bin Rashid University of Medicine and Health Sciences, Dubai, United Arab Emirates; ^14^Department of Anatomy, College of Medicine, Mohammed Bin Rashid University of Medicine and Health Sciences, Dubai, United Arab Emirates; ^15^Department of Surgery, College of Medicine and Health Sciences, United Arab Emirates University, Al Ain, United Arab Emirates; ^16^Department of Surgery, Tawam Hospital, Abu Dhabi, United Arab Emirates; ^17^Department of Radiology, Tawam Hospital, Abu Dhabi, United Arab Emirates; ^18^Department of Physiology, College of Medicine and Health Sciences, United Arab Emirates University, Al Ain, United Arab Emirates

**Keywords:** blended machine learning model, deep learning, COVID-19, pneumonia, SARC-CoV-2, lung structural changes, structure-function association, hypoxia

## Abstract

**Background:**

Hypoxia is a potentially life-threatening condition that can be seen in pneumonia patients.

**Objective:**

We aimed to develop and test an automatic assessment of lung impairment in COVID-19 associated pneumonia with machine learning regression models that predict markers of respiratory and cardiovascular functioning from radiograms and lung CT.

**Materials and Methods:**

We enrolled a total of 605 COVID-19 cases admitted to Al Ain Hospital from 24 February to 1 July 2020 into the study. The inclusion criteria were as follows: age ≥ 18 years; inpatient admission; PCR positive for SARS-CoV-2; lung CT available at PACS. We designed a CNN-based regression model to predict systemic oxygenation markers from lung CT and 2D diagnostic images of the chest. The 2D images generated by averaging CT scans were analogous to the frontal and lateral view radiograms. The functional (heart and breath rate, blood pressure) and biochemical findings (SpO_2_, $HCO_{3}^{-}$, *K*^+^, *Na*^+^, anion gap, C-reactive protein) served as ground truth.

**Results:**

Radiologic findings in the lungs of COVID-19 patients provide reliable assessments of functional status with clinical utility. If fed to ML models, the sagittal view radiograms reflect dyspnea more accurately than the coronal view radiograms due to the smaller size and the lower model complexity. Mean absolute error of the models trained on single-projection radiograms was approximately 11÷12% and it dropped by 0.5÷1% if both projections were used (11.97 ± 9.23 vs. 11.43 ± 7.51%; *p* = 0.70). Thus, the ML regression models based on 2D images acquired in multiple planes had slightly better performance. The data blending approach was as efficient as the voting regression technique: 10.90 ± 6.72 vs. 11.96 ± 8.30%, *p* = 0.94. The models trained on 3D images were more accurate than those on 2D: 8.27 ± 4.13 and 11.75 ± 8.26%, *p* = 0.14 before lung extraction; 10.66 ± 5.83 and 7.94 ± 4.13%, *p* = 0.18 after the extraction. The lung extraction boosts 3D model performance unsubstantially (from 8.27 ± 4.13 to 7.94 ± 4.13%; *p* = 0.82). However, none of the differences between 3D and 2D were statistically significant.

**Conclusion:**

The constructed ML algorithms can serve as models of structure-function association and pathophysiologic changes in COVID-19. The algorithms can improve risk evaluation and disease management especially after oxygen therapy that changes functional findings. Thus, the structural assessment of acute lung injury speaks of disease severity.

## 1. Introduction

The outbreak of the coronavirus disease 2019 (COVID-19) resulted in a steady rise in the number of confirmed cases and excess mortality (1). Due to the pandemic, the need for noninvasive respiratory support of patients with severe pneumonia became overwhelming and often exceeded healthcare capacity. This also necessitated the development of methods for improving patient monitoring (2). At the time when the disease resulted in a high death rate especially in the elderly population, researchers created machine learning (ML) models to stratify risks for the proper resource allocation (3–5). The utility of these models in clinical settings was limited as they did not provide a quantitative metric of disease severity while forecasting outcomes. For this reason, it was challenging to assess the level of lung impairment and the models built were not applicable to patients with other types of community-acquired pneumonia (CAP).

Currently, COVID-19 has spread around most of the world and it has been elucidated that the risk of severe disease is relatively low in healthy individuals especially children and young adults (6, 7). Instead, there is a high number of mild and moderate cases to follow. The vast majority of cases experience mild and moderate disease compared to severe disease, and most patients have a favorable prognosis. However, severe hypoxemia secondary to SARS-CoV-2 infection can lead to acute respiratory failure (8). The prognosis of severe disease course is more accurate when a multidisciplinary approach is used (9). Physicians need models that would describe pathophysiologic changes and alterations of the lung parenchyma during the disease course. Moreover, information on the correlation between structural changes and the functional consequences is insufficient even for CAP, let alone SARS-CoV-2 associated pneumonia (SAP). Developing models of structure-function association would improve a routine radiologic assessment which is currently based on the visual evaluation of the percentage of consolidation and opacification within the total lung volume. In addition to the visual assessment, we aim to develop and test a tool to evaluate the level of lung damage in pneumonia by predicting the level of hypoxia that would be observed if no treatment is provided.

### 1.1. Hypoxia in Viral and Bacterial Pneumonia

Hypoxia is a potentially life-threatening condition that can be seen in patients with either atypical viral pneumonia or bacterial pneumonia due to severe lung compromise (10). Over 30% of SARS-CoV-2 infected patients without shortness of breath might present with hypoxemia on admission (11). At the beginning of the pandemic, information on the difference between bacterial pneumonia and COVID-19-associated pneumonia was missing. In addition, we did not have data on the hypoxia features specific to this type of atypical pneumonia. The impulsive use of lung mechanical ventilation in severe disease forms ended up in approximately 88% fatality (12).

The mechanisms of developing hypoxia and its structural correlates merit medical attention as their understanding would aid in choosing the proper therapeutic strategy and risk management. **In viral pneumonia**, pathogen proliferation results in *alveolar inflammation* and *reduced surfactant production* both leading to pneumonic consolidation and consequent hypoxia. **In SAP**, another potential mechanism contributing to hypoxia is *pulmonary vasoconstriction* (10). **Pathogenesis of bacterial pneumonia** is mediated by a pulmonary defense system that consists of alveolar macrophages and other immune cells which engulf bacteria and produce inflammation. Cytokines are responsible for leakage of the alveolar-capillary membrane thus causing hypoxia (13). Contrarily to the bacterial pneumonia, most patients with COVID-19 display low circulating lymphocyte counts, and a decrease in the number of T lymphocytes and their subtypes correlate with disease severity (14). The variety in the immune response may result in distinct functional outcomes.

### 1.2. Dissociation Between Structural and Functional Changes

Hypoxia is an outcome of substantial endothelial damage which can be more pronounced in COVID-19 than in other viral pneumonias. Within a year of active research on COVID-19, specialists managed to report distinct patterns of relationship between the lung structure and the functional outcomes of the disease. *At an early stage* of COVID-19, demolition of the lung may predominate clinical severity of cases. The clinical appearance of the disease is relatively mild in contrast to the gravity of the radiologic findings. This fact serves as an argument in favor of early administration of corticosteroid drugs that downregulate the hyperimmune response and prevent disease progression (8). *At a late stage*, the situation commonly reverses. Hypoxia can worsen disproportionally to the lung involvement. A possible explanation for such a discrepancy is a disruption of the structure-function association because of the disregulation of lung perfusion. The disruption may vary throughout the disease course, causing different phenomena specific for COVID-19. Considering different diagnostic modalities improves the accuracy of clinical assessment and justifies the advantage of a multidisciplinary diagnostic approach involving specialists in pulmonology, radiology, and pathology (2, 9).

#### 1.2.1. Silent Hypoxemia in COVID-19

Physicians reported that COVID-19 pneumonia caused oxygen deprivation which was difficult to detect since the patients did not experience shortness of breath (15). The condition was termed as “silent” hypoxia (16). The virus caused a collapse of the alveoli: it did not fill them with fluid or pus as in CAP. The still-efficient removal of carbon dioxide hid the clinical appearance of hypoxia in the initial stages of COVID-19 pneumonia (15). Abnormal gas exchange results from the known physiology of viral pneumonias and acute respiratory distress syndrome (ARDS). The elevated ventilation improves elimination of carbon dioxide, whereas oxygenation rises to a lesser extent (17). This happens because of the intrapulmonary shunt and ventilation-perfusion (V/Q) mismatch. In this way, hypoxic suppression of dyspnea reduces the manifestation of symptoms, and profound hypoxemia can remain unnoticed until exertion.

#### 1.2.2. Atypical (Severe) Form of Acute Respiratory Syndrome in COVID-19

Though COVID-19 pneumonia meets diagnostic criteria of ARDS, the patients infected with SARS-CoV-2 present with an uncommon form of the syndrome. A hypothesis explains severe hypoxemia in COVID-19 with hypoxic vasoconstriction. A drop in oxygen saturation below some threshold leads to overaccumulation of byproducts of hypoxia; this creates a vicious circle of hypoxic changes and causes a loss of lung perfusion regulation as an outcome (18). So, the cardio-respiratory compensation to hypoxemia achieved by the patients initially may fail precipitously (17).

### 1.3. Studying Structure-Function Association in Patients With ARDS

Alveolar filling as a central feature of ARDS has both structural and functional outcomes. To elucidate structural changes, physicians resort to *lung radiographs*. They use markers of *impaired gas exchange* as physiological metrics. These methods are universal disregarding the reason for the fluid accumulation within alveoli which may differ in ARDS of distinct etiology (inflammation mediated alveolar fluid leak in COVID-19 vs. elevated transcapillary pressure in CAP with high-altitude pulmonary edema) (17). Because of these features, structure-function association in the compliant lung may also vary between the pathologies.

To prioritize resources for COVID patients effectively, physicians resort to clinical markers of COVID-19 severity. The markers can be classified into (i) *the laboratory findings* describing biochemical and hematologic shifts; (ii) *the structural data* depicting morphologic abnormalities [e.g., chest X-ray (CXR), CT acquistions, bronchoscopy, etc.]; and (iii) *the functional features* showing breath and heart beat rate at rest and after physical load.

*Laboratory findings*. Recently we used machine learning for processing laboratory findings of the patients with SARS-CoV-2 to rank the biomarkers of disease severity and to build the prediction model of the disease course (3). The performance of the neural network trained with top valuable features (aPTT, CRP and fibrinogen) was admissible (area under the curve (AUC) 0.86; 95% CI 0.486 to 0.884; *p* < 0.001). The laboratory results may serve as predictors of the disease progression as some of them reflect the degree of inflammation and the others represent unmanaged chronic conditions that increase the risk of complications (3).

*Morphological findings*. Pulmonary inflammation is associated with the clinical symptoms and the laboratory findings (19). The degree of the inflammatory process is typically assessed with the radiologic findings. Examination of chest radiograms (CXR) and lung CT reflects the level of the structural impairment of the lung parenchyma and the supposed outcomes. The CT findings differ by the stage of the disease: ground-glass opacity dominate in the early COVID-19 followed by crazy paving and consolidation later in the disease course (20, 21).

There is evidence that both chest radiography and laboratory findings are important for assessing the severity of the disease (20), and it is feasible to establish an accurate prediction model of the outcome of SARS-CoV-2 pneumonia based on either type of data (22, 23). The laboratory and the morphological findings are supposed to be more predictive if used in combination (24). Unfortunately, the predictive models built on either type of the clinical data had some limitations that precluded their use in clinical practice (25).

## 2. Objectives

We focused on the association between lung CT findings and the functional status of COVID-19 patients. The principal aim of the study was to develop and test a tool for automatic assessment of lung impairment in COVID-19 associated pneumonia with machine learning (ML) regression models that predict markers of respiratory and cardiovascular functioning from radiograms and lung CT. We utilized chest CT images reconstructed with distinct kernels and pre-processed to obtain either 2D or 3D images of extracted or non-extracted lung as the morphological findings. The markers of respiratory and cardiovascular functioning reflected the level of hypoxia in pneumonia patients.

*Hypothetically*, the diagnostic value of multi-detector row CT is sufficient to predict the functional outcomes of the injury to the lung in SAP. By testing various approaches we expected to find a combination of optimal settings for reconstructing and pre-processing diagnostic images of the lung for machine learning. The settings would help us to reliably build the models reflecting the patient's functional status from medical images. The analysis with advanced statistical methods would help the physicians to compare follow-up studies, detect disease worsening, and stratify risks thus improving patient management and outcomes.

To address the objectives, we formulated the following subobjectives:

1. Study the associations of the radiologic estimates of lung injury with biochemical and physiological markers of hypoxia.
2. Compare the predictive potential of single- vs. multiplanar 2D diagnostic images to reflect the level of systemic oxygenation.
3. Build 3D models of structure-function association and estimate the boost in performance after extraction of the lungs from 3D images.
4. Select the optimal reconstruction kernel for the diagnostic images of the lung with regard to the predictive potential of ML models fed with the images.

## 3. Materials and Methods

### 3.1. Study Sample

All the patients consecutively admitted to Al Ain Hospital, Abu Dhabi Emirate from 24 February to 1 July, 2020 were enrolled into the study. At that time *The National Guidelines of the management of the patients with COVID-19* required that everybody who tested positive for SARS-CoV-2 was hospitalized irrespective of disease severity (e.g., presenting any symptoms). Physicians of the hospital observed many mild and asymptomatic forms of the disease. Before the treatment started, all the required spectrum of analyses were conducted on the day of admission. These included lung CT; measurement of oxygen saturation by pulse oximetry (SpO_2_); physical examination and assessment of the complete blood count, level of electrolytes and C-reactive protein (CRP).

In this study we did a retrospective analysis of a unique dataset of cases. It reflected the full range of disease forms (from mild to critical) at the early phase of the disease. All the cases occurred at the time when β-variant of COVID-19 was the predominant variant in the UAE and in most other countries. The dataset comprises demographic features (age, sex), the functional data [heart rate (HR), breath rate (BR), systolic (SBP) and diastolic blood pressure (DBP)], hematological and biochemical findings, data on SpO_2_, radiological examinations (lung CT) of the patients *on admission*. The inclusion criteria for our study were as follows: age 18 years or older; inpatient admission; SARS-CoV-2 positive real-time reverse-transcriptase polymerase chain reaction (PCR) from nasopharyngeal swabs only; full DICOM lung CT examination available at PACS. A total number of 605 cases met the criteria.

#### 3.1.1. Pulse Oximetry, Physical Examination and Measurement of Laboratory Findings

Follow clinical practice guidelines. *Anion gap (AG)* was calculated from the concentration of the major ions (*Na*^+^, *K*^+^, *Cl*^−^, and $HCO_{3}^{-}$) in serum expressed in *mmol*/*l* (see Formula 1).


(1) $$\begin{array}{r} AG=\left(\left[Na^{+}\right]+\left[K^{+}\right]\right)-\left(\left[Cl^{-}\right]+\left[HCO_{3}^{-}\right]\right) \end{array}$$

#### 3.1.2. CT Scanning and Reconstructing Settings

The high-resolution CT scan protocol was as follows: the tube voltage 120 kV, the electric current 195 mA, the exposure time 0.5 s and the slice thickness 1mm. The scanning range was from lung apex to diaphragm in the axial plane taken at the end of inspiration. We applied three reconstruction filters (kernels) of distinct smoothness (B30f, B60f and B80f) for the reconstruction of CT images. We assured the accuracy of Hounsfield unit (HU) in the CT scanner with a standard water phantom. Since the severity of the lung involvement on the CT correlated with the severity of the disease, we calculated the total lung CT score and the percentage of lung involvement. *The total CT score* is a semi-quantitative score of pulmonary involvement, and it rates the percentages of each of the five lobes that is injured: <5%, 5–25%, 26–49%, 50–75%, and > 75% involvement (26). The total CT score is the sum of the individual lobar scores and can range from 0 (no involvement) to 25 (maximum involvement) when all five lobes show more than 75% involvement. We calculated the CT score in the automatic way described in Section 3.4.

### 3.2. Pre-processing of the Data

In our dataset, the voxel intensities ranged from –1,024 to over 2,000 HU. First, all images underwent the intensity correction by changing the values of voxels below –1,000 and above 400 to –1,000 and 400, respectively. Then voxel intensity (*v*) of images was normalized with the min-max scaling technique as below:


(2) $$\begin{array}{r} v=\frac{v-v_{min}}{v_{max}-v_{min}} \end{array}$$

We also segmented the lungs, performed the background removal and cropped them within the lung boundaries. Finally, full and extracted lung images were resized to 128 x 128x 64 voxels (3D-nonextracted and 3D-extracted datasets were created).

To reduce the data complexity and to test the predictive power of Deep Learning (DL) models, we also created several two-dimensional datasets. With *X*, *Y*, and *Z* variables denoting the dimensions of the CT image in axis *x*, *y*, and *z*, CT scan were described in the following way:


(3) $$\begin{array}{r} I=\left\{\left(v_{x},v_{y},v_{z}\right):x=\bar{1,X},y=\bar{1,Y},z=\bar{1,Z}\right\} \end{array}$$

The *j*^*th*^ anterior-posterior (coronal), side (sagittal) and transversal (axial) slices of the image *I* were defined as:


(4) $$\begin{array}{l} s_{sagittal}^{\left(j\right)}=(j,v_{y},v_{z})\\ s_{coronal}^{\left(j\right)}=(v_{x},j,v_{z})\\ \;s_{axial}^{\left(j\right)}=(v_{x},v_{y},j) \end{array}$$

The corresponding averaged images were determined as follows:


(5) $$\begin{array}{l} I_{sagittal}=\frac{1}{X}\sum_{i=1}^{X}s_{sagittal}^{\left(i\right)}\\ I_{coronal}=\frac{1}{Y}\sum_{i=1}^{Y}s_{coronal}^{\left(i\right)}\\ \;I_{axial}=\frac{1}{Z}\sum_{i=1}^{Z}s_{axial}^{\left(i\right)} \end{array}$$

We generated a dataset of 2D diagnostic images from the 3D ones (CT findings). The reason why we did not resort directly to CXR were as follows. *First*, the study had a retrospective design and the patients were scanned only with CT. Evidently, the physicians did not order CXR as a separate examination to avoid an additional exposure of the patients to radiation without diagnostic benefits. *Second*, the fact that diagnostic images were acquired with one machine for the same study cohort ensures the valid comparison of the performance of the models trained on the 2D and 3D datasets. The 2D and 3D diagnostic images differ only in the diagnostic value with no confounders impacting the final diagnostic quality (e.g., different brands, settings, dosage, etc.).

**Figure 1:**
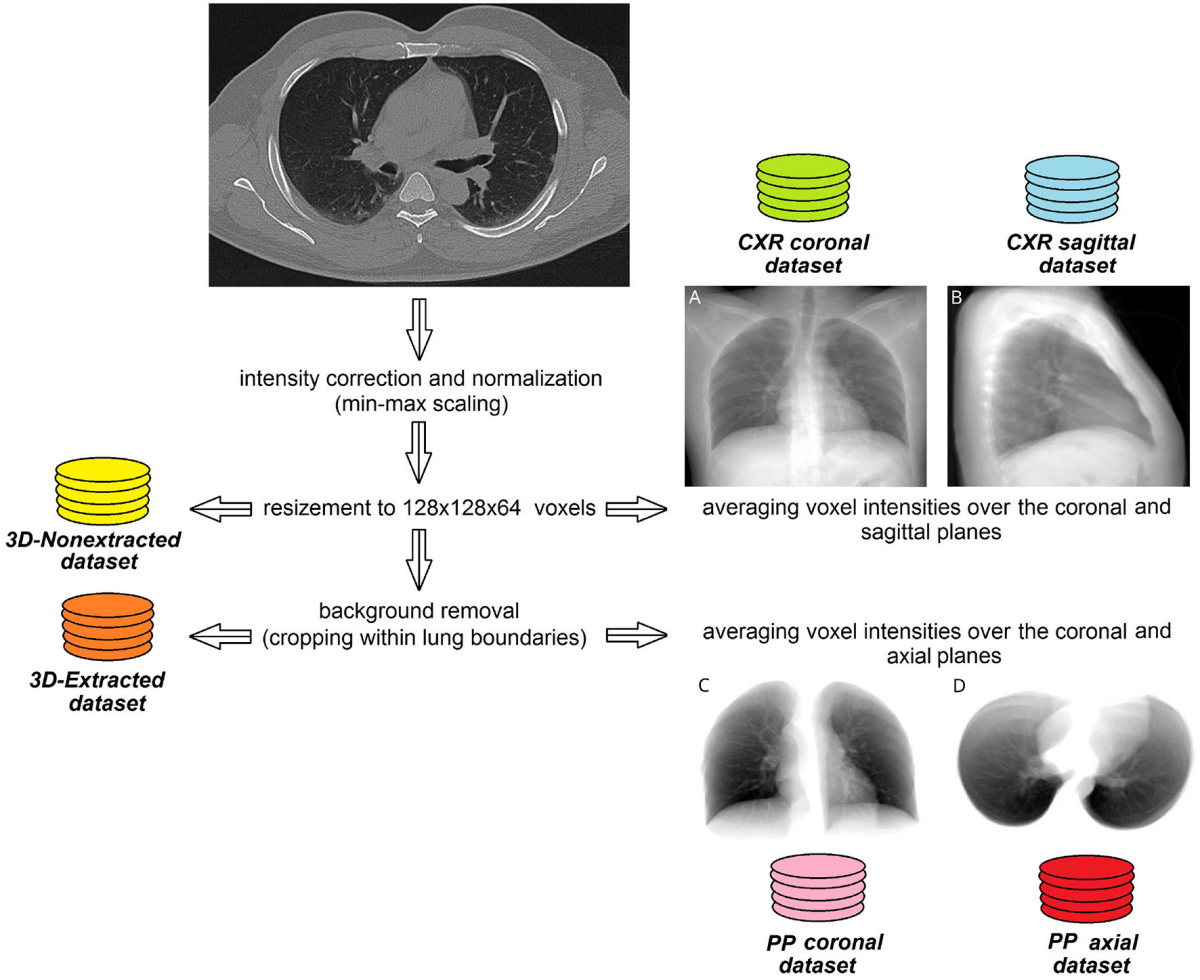
Steps of pre-processing lung CT images to create 2D and 3D datasets. Sample images averaged over coronal **(A)** or sagittal **(B)** plane without background removal. Same case, images averaged over coronal **(C)** or axial **(D)** plane with lung extraction.

We averaged voxel intensities over the coronal and sagittal planes in the 3*D*−*nonextracted* dataset and over the axial and coronal planes in the 3*D*−*extracted* dataset. In this way we created four additional two-dimensional datasets *CXR*_*coronal*_, *CXR*_*sagittal*_, *PP*_*axial*_, and *PP*_*coronal*_, respectively. Finally, all 2D lung images were resized to 250 by 150 pixels utilizing down-sampling with nearest-neighbor interpolation and stored in JPEG format as illustrated in Figure 1.

### 3.3. 2D and 3D CNN Models

To predict the biochemical and functional markers of the disease severity, we designed two CNN models, 2D- and 3D CNN. The first model was built to be trained on pre-processed 2D-images (see Section 3.2). We assembled the model in the following way. We utilized EfficientNetB4 model pre-trained on Imagenet dataset (27, 28). The top flatten layers were excluded from the model and substituted with three consecutive fully-connected layers of 256, 64, and 32 neurons. The designed model was trained in end-to-end manner using the five-fold cross-validation technique and RMSprop optimizer with the initial learning rate of 0.001 and ρ = 0.9. In each fold 10% of the training subset were used for validation. We resorted to the mean absolute error (MAE) to evaluate the loss while training as the output variables were continuous. The model was trained with 150 epochs or till the validation loss dropped across 20 consecutive epochs. The predicted values at each epoch were combined to report the averaged value of MAE. The final performance was calculated as the fraction of MAE to the range of values (*MAE*/*range*, %).

The second model was based on 3D CNN architecture. It was fed with 3D-dimensional images 128 x 128 x 64 in size. The model consisted of three 3D convolutional layers of the size of 64, 128, and 256 units, respectively. Each layer was followed by the max-pooling block. Then we allocated a global averaged pooling block followed by a fully connected dense layer of 512 units. At last, we applied a dropout function of the rate of 30% to the weights of the layer. We set Adam optimizer parameters for the following values: the learning rate = 0.0001, β_1_ = 0.9, β_2_ = 0.999, ϵ = 1*e*−08, *decay* = 0.0). The major part of the dataset (70%) was used for training purposes. 30% of the training data were utilized to validate the results. The remaining part of the dataset was allocated for testing. The designed model was trained either till 150 epochs or till the validation loss did not drop across 20 consecutive epochs. We employed the augmentation technique to train the model. Before feeding the model, the images were rotated by the angle from –25° to 25° with the step of 5°. The final performance was calculated as the fraction of *MAE*/*range*, %.

### 3.4. Methodology of the Study

**Figure 2:**
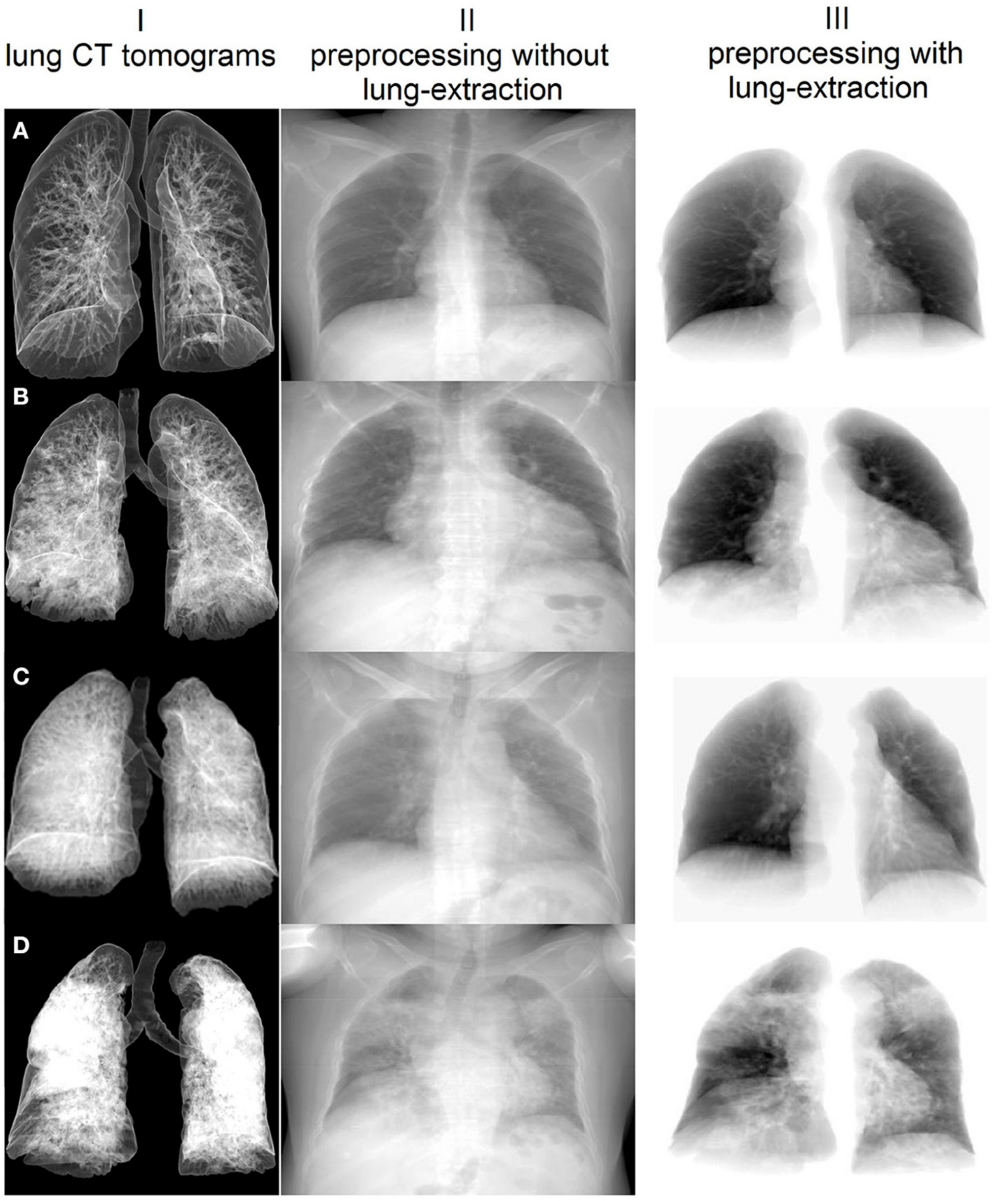
Sample images extracted with the proposed pre-processing steps and averaged over the coronal plane of the lung CT examination. I – Lung CT presented with volume rendering technique; different percentages of lung involvement: **(A)** 1.16%, **(B)** 15.83%, **(C)** 29.09%, **(D)** 62.29%. Pre-processed 2D images with (II) and without background (III).

*Working on subobjective one*, we estimated associations between the biochemical (AG, serum potassium, HCO_3_, CRP), the functional (HR, BR, SBP, DBP, SpO_2_) and the radiological (total CT score, percentage of lung involvement) markers of disease severity by calculating Pearson's correlation coefficients. We also added age as a significant risk factor to the correlation matrix. We assessed the total lung CT score and the percentage of lung involvement from the radiologic markers. First, the lungs and lung lobes were segmented with the help of DL U-net model trained on a large and diverse dataset described in Hofmanninger et al. (29). See samples of the extracted and non-extracted images in Figure 2. Masks of the lungs and lobes were stored for further analysis. Second, we segmented the lesions with CT Thorax COVID-19 model from MedSeg tool (30). The model was trained on a dataset segmented manually by radiologists. The output of the model was masks of lesions of the following types: ground-glass opacity (GGO), consolidation and pleural effusion. We utilized fslstats tool from FSL to calculate the volume of lesions (31). This allowed us to determine the absolute value and *the percentage of the involvement* for the entire lung and for each lung lobe. Finally, we calculated *the total CT score (CTS)* by summing up the score for the involvement of each lung lobe (1 for <5%, 2 for 5–25%, 3 for 26–49%, 4 for 50–75%, and 5 for > 75%).

**Figure 3:**
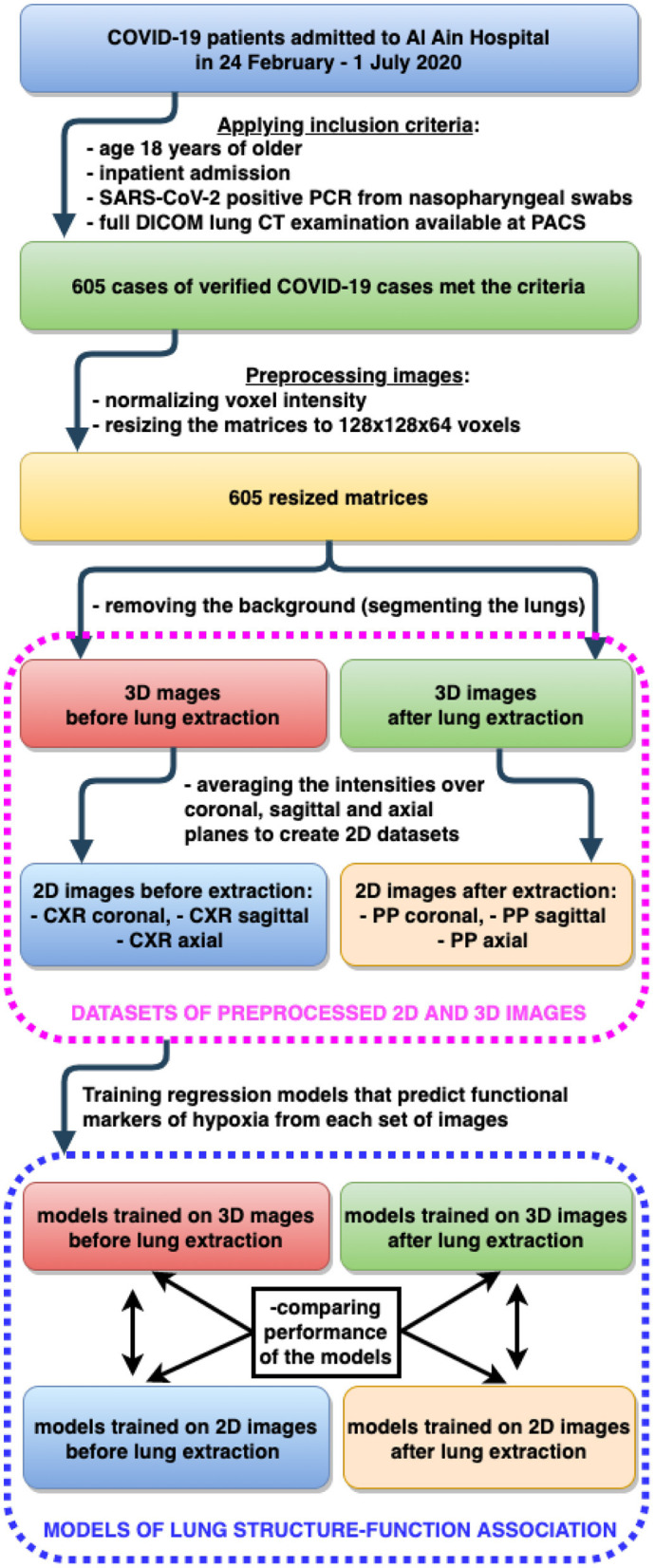
Building machine learning regression models to predict functional markers of hypoxia from 2D and 3D diagnostic images of lung.

See Figure 3 for details on building machine learning models to fulfill subobjectives 2–4. *The rationale behind subobjective two* was to receive more information on the studied structure by examining the diagnostic images in several planes. The approach is analogous to the method of obtaining a side view radiogram in addition to the frontal one. We designed a CNN-based regression model to predict the markers of systemic oxygenation from 2D diagnostic images of the chest. We used functional (HR, BR, SBP, DBP) and biochemical findings (SpO_2_, serum potassium level and AG) as the laboratory markers of disease severity. A metric of the final performance of the models was a proportion of the mean averaged error to the range of values (*MAE*/*range*, %). We trained the designed DL model on images from *CXR*_*coronal*_, *CXR*_*sagittal*_, *PP*_*axial*_, and *PP*_*coronal*_ datasets (see Section 3.2). At the input some models had 2D diagnostic images reconstructed in a single plane (coronal, sagittal or axial), the others received 2D radiograms in two planes (coronal and sagittal, axial and coronal), see Figure 1. We used two architectures for radiograms acquired in two planes: Data Blending (DB) and Voting Regression (VR). The first approach is multimodal: the model is fed with two images. The second solution is an ensemble model in which each comprising algorithm is fed with a single image.

*To address the third subobjective*, we designed 3DCNN regression model and trained it on 3D-Nonextracted and 3D-Extracted datasets separately. The quality of the model output was measured in the same way as described above (*MAE*/*range*, %). We employed the Mann-Witney U test to estimate the boost of performance due to information noise reduction after the lung extraction.

*To reach the goal of the final subobjective*, we trained the CNN-regression model on the images from three different kernels individually. We used the Kruskal-Wallis test to compare the results of B30f, B60f and B80f kernel images regarding noise as a function of the reconstruction kernel. The kernels were ranked according to the predictive power of the DL models.

In this study, DL models were trained and tested using the five-fold cross-validation technique. Ten percent of the training data in each fold were used for validation. We employed early stopping callback functions to monitor the loss at the validation set.

### 3.5. Hardware and Software Used

We employed a 40 CPU core Linux Ubuntu 18.04 Nvidia DGX-1 deep learning server equiped with 32 GB 8 NVIDIA Tesla V100 GPU. The server had a web-based multi-user concurrent job scheduling system (32). The experimental work was conducted using Python and its libraries for DL, Data Processing, and Data visualization, such as tensorflow-gpu v.2.3.1, keras v.2.4.3, SciPy v.1.16.4, NumPy, Pandas, Matplotlib, Seaborn. We installed Neurodocker which wraps up the aforementioned software in a complete file system (33).

## 4. Results

### 4.1. Associations Between Radiologic Estimates of Lung Injury, Biochemical and Physiological Markers of Hypoxia

**Figure 4:**
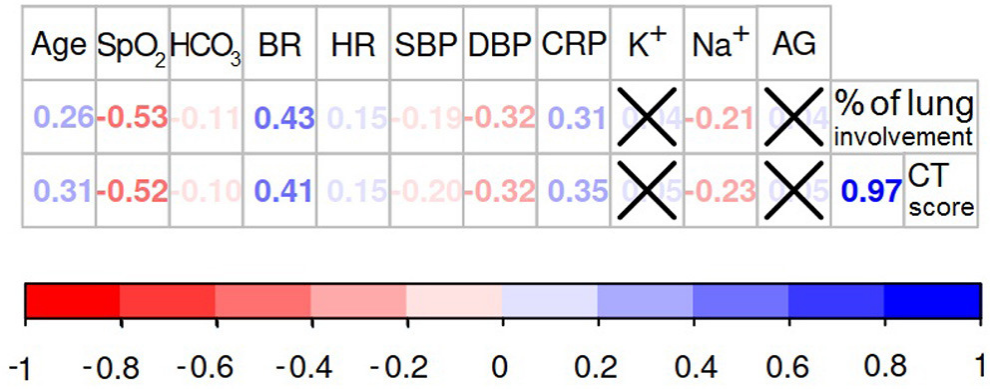
Association between structural findings of the lung impairment and the functional markers of oxygen deprivation. If the association between variables is significant (*p* < 0.05) the values of Spearman's rank correlation coefficients are presented in the diagram, otherwise the values are crossed out.

To justify the accuracy of the collected data, we built a correlation matrix displaying the correlation coefficients for (a) age—the major individual risk factor, (b) radiologic markers of lung involvement and (c) functional and biochemical finding—laboratory markers of disease severity (see Figure 4).

The percentage of the lung involvement strongly correlated with the CT score (*r* = 0.97; *p* < 0.001). Both metrics of the lung structural changes were intimately associated with the majority of the physiological and biochemical markers of the oxygen deprivation. Relationships between these findings express the close association between the structural and functional data. Obviously, lung parenchymatous inflammation has a detrimental effect on its ventilatory function. Oxygen saturation level showed the most significant correlation with the percentage of the lung involvement (*r* = −0.53; *p* < 0.001) and the CT score (*r* = −0.52; *p* < 0.001). Less strong association was found between SpO_2_ level and cardiovascular system parameters: breath rate (*r* = −0.58; *p* < 0.001), heart rate (*r* = −0.14; *p* = 0.001), systolic (*r* = 0.26; *p* < 0.001) and diastolic blood pressure (*r* = 0.38; *p* < 0.001). These observations provide evidence for the “silent hypoxia” phenomenon described in Section 1.2.

We did not find a direct correlation between the serum level of potassium, AG and the level of the lung deterioration assessed with CT score (K^+^: *r* = 0.05; *p* = 0.224; AG: *r* = 0.05; *p* = 0.242) or the percentage of lung involvement (K^+^: *r* = 0.04; *p* = 0.306; AG: *r* = 0.05; *p* = 0.312). Noticeably, the SpO_2_ level was not associated with AG (*r* = −0.03; *p* = 0.359). These findings can indicate the presence of non-metabolic respiratory acidosis in COVID-19 pneumonia. However, the metabolic factors correlated with some physiological parameters. For instance, the AG level showed weak but significant correlations with HR (*r* = 0.12; *p* = 0.003), SBP (*r* = 0.15; *p* = < 0.001) and DBP (*r* = 0.16; *p* = < 0.001). A weak significant association was observed between HCO_3_ level and SBP (*r* = −0.11; *p* = 0.008), heart and breath rate (HR: *r* = −0.19; *p* ≤ 0.001; BR: *r* = −0.14; *p* = < 0.001). These observations may illustrate some metabolic compensation for cardiovascular functioning. Contrarily, the serum potassium concentration was not associated with any of the tested cardiovascular parameters. This lack of association could possibly be explained by the background diseases which may also account for the electrolyte imbalace.

### 4.2. Single- vs. Multiplanar 2D Diagnostic Images

#### 4.2.1. Single-Planar 2D Diagnostic Images

The sagittal view radiograms, if fed to ML models, reflected dyspnea more accurately than the coronal view radiograms. One potential reason for this is their smaller size and the lower model complexity. However, the difference in accuracy was not pronounced (*p* = 0.28). MAE/range was 10.43 ± 5.40 for *CXRcoronal* vs. 13.49 ± 11.81% for *CXRsagittal* (see Table 1). Based on the study findings, it seems that the model trains in a better way because the size of the sagittal reconstruction is smaller than in other projections. However, the right and left lungs overlap in this view.

**Table 1:** Performance of the CNN-based regression models trained on the 2D datasets in terms of MAE/range,%.

**Data**											
	**SpO_2_**	**BR**	**HCO_3_**	**CRP**	**K**	**Na**	**AG**	**HR**	**SBP**	**DBP**	**Mean ± SD**
**B30f**											

CXR coronal(C)	6.030	4.630	5.460	15.170	34.770	13.990	6.610	17.430	10.390	11.790	12.627 ± 8.959
CXR sagittal (S)	10.01	4.060	4.890	14.040	22.920	11.500	4.450	14.620	8.830	10.910	10.623 ± 5.748
CXR DB(C+S)	10.5	3.780	5.090	13.160	31.100	15.750	4.830	16.570	8.190	12.440	12.141 ± 8.059
CXR VR(C+S)	8.02	4.345	5.175	14.605	28.845	12.745	5.530	16.025	9.610	11.350	11.625 ± 7.264
PP coronal (C)	8.06	4.030	5.530	13.610	20.840	12.210	4.550	16.080	8.560	10.570	10.404 ± 5.398
PP axial (A)	12.02	4.070	5.420	13.460	29.490	9.130	4.400	15.080	8.540	11.160	11.277 ± 7.432
PP DB(A + C)	12.66	3.760	5.060	13.040	16.790	10.280	4.470	16.580	8.300	10.460	**10.140** **±4.745**
PP VR(A + C)	10.04	4.050	5.475	13.535	25.165	10.670	4.475	15.580	8.550	10.865	10.841 ± 6.492
Average	9.668	4.091	5.263	13.828	26.240	12.034	4.914	15.996	8.871	11.193	11.21 ± 6.582
**B30f - 3D images**											

3D-Nonextracted	8.404	3.128	3.358	3.441	13.317	12.376	5.808	13.394	9.199	10.287	8.271 ± 4.13
3D-Extracted	6.124	2.946	3.21	3.452	13.217	11.825	5.895	13.273	9.137	10.345	**7.941** **±4.131**
**B60f**											

CXR coronal(C)	6.480	4.390	5.430	15.340	37.330	15.410	5.800	16.290	9.600	12.790	12.886 ± 9.726
CXR sagittal(S)	5.530	3.970	4.840	13.690	21.560	12.010	5.290	15.890	8.580	11.670	**10.303** **±5.725**
CXR DB(C+S)	6.980	3.920	5.230	13.440	27.020	9.700	5.220	14.470	8.340	11.480	10.58 ± 6.789
CXR VR(C+S)	6.005	4.180	5.135	14.515	29.445	13.710	5.545	16.090	9.090	12.230	11.595 ± 7.624
PP coronal(C)	10.54	5.030	5.320	15.360	23.310	13.280	4.770	15.480	8.960	10.550	11.26 ± 5.835
PP axial(A)	13.79	3.820	5.440	12.700	22.570	11.300	6.010	17.870	8.720	11.600	11.382 ± 5.805
PP DB(A+C)	7.420	3.950	4.750	13.570	35.180	13.110	6.020	15.000	8.130	10.890	11.802 ± 9.067
PP VR(A+C)	12.165	4.425	5.380	14.030	22.940	12.290	5.390	16.675	8.840	11.075	11.802 ± 5.744
Average	8.614	4.211	5.191	14.081	27.419	12.601	5.506	15.971	8.783	11.536	11.391 ± 6.878
**B80f**											

CXR coronal(C)	5.78	4.620	5.580	15.670	60.000	14.710	5.510	16.340	9.360	12.130	14.97 ± 16.462
CXR sagittal(S)	6.05	3.990	5.240	13.950	19.920	13.310	4.930	15.160	9.760	11.550	10.386 ± 5.298
CXR DB(C+S)	6.3	4.230	5.700	13.010	22.460	9.130	3.960	13.850	9.900	11.350	**9.989** **±5.614**
CXR VR(C+S)	5.915	4.305	5.410	14.810	39.960	14.010	5.220	15.750	9.560	11.840	12.678 ± 10.513
PP coronal(C)	6.100	4.010	5.670	13.630	32.610	12.610	4.890	17.020	8.490	10.810	11.584 ± 8.526
PP axial(A)	11.93	3.910	4.750	13.140	22.050	12.020	6.300	15.470	8.320	10.960	10.885 ± 5.447
PP DB(A+C)	11.98	4.220	4.780	14.660	27.300	12.160	4.320	17.090	8.710	11.670	11.689 ± 7.061
PP VR(A+C)	9.015	3.960	5.210	13.385	27.330	12.315	5.595	16.245	8.405	10.885	11.235 ± 6.864
Average	7.884	4.156	5.293	14.032	31.454	12.533	5.091	15.866	9.063	11.399	11.677 ± 8.007

*A, S, C correspond to averaged lung CT image in appropriate plane; VR, Voting Regression meta-estimator; MB, Model Blending; DB, Data Blending*.

#### 4.2.2. Multiplanar 2D Diagnostic Images

Table 1 shows that multiplane reconstruction boosts the performance of the models that were initially trained with 2D data in a single reconstruction plane. The accuracy of the models trained on single-projection radiograms was around 11÷12% and it dropped by 0.5÷1% if both projections were applied: 11.97 ± 9.23 (*CXRcoronal* and *CXRsagittal*) vs. 11.43 ± 7.51% (*CXR* *DB* and *CXR* *VR*). Thus, the ML regression models based on 2D images acquired in multiple planes showed slightly better performance (*p* = 0.70). The diagnostic images acquired in the sagittal plane contribute additional information to the ones in the coronal view. The latter shows a typical chest radiogram in the anteroposterior or reverse projection (see Figure 1A) while the sagittal reconstruction is analogous to the lateral projection (see Figure 1B). Radiologists can make more accurate assumptions on the location and the spread of the lung lesions by combining the frontal and lateral views. We expected to achieve the same result with machine learning. However, the observed improvement in performance was not statistically significant (from over 11÷12% by 0.5÷1%).

The data blending approach was as efficient as the voting regression technique: 10.90 ± 6.72 vs. 11.96 ± 8.30%, *p* = 0.94 (all *CXR* *DB* vs. all *CXR* *VR*). There was no marked difference in accuracy between these architectures. We had expected that the model fed with images acquired in two mutually perpendicular planes would be more accurate than the ensemble solution. This did not happen because of the rise in computational complexity of the algorithm that analyzes both coronal and sagittal view radiograms.

### 4.3. Accuracy of 3D Models and Results of Applying Lung Extraction Technique

We assessed the performance of regression models predicting markers of systemic oxygenation from 3D to 2D diagnostic images of the chest. We did it before and after lung extraction (see Figures 1A–D).

#### 4.3.1. Reduction of the Information Noise With a Lung Extraction Technique

We cropped the images to extract the lungs. In this way we tried to reduce the information noise coming from the tissues outside of the lungs. *In 2D models*, training the models on the extracted lung images resulted in only marginally better performance than feeding them with the radiograms that contain background (e.g., ribs, vertebral column, etc.). Furthermore, the most accurate prediction was achieved using the data blending models trained on the non-segmented lung images averaged in coronal and sagittal plane (*MAE*/*range* = 9.989 ± 5.614%). For the models based on coronal-view radiograms MAE/range was 13.49 ± 11.81 in *CXRcoronal* before the lung extraction and it dropped to 11.08 ± 6.51% in *PPcoronal* after the lungs were cropped (*p* = 0.54). *In 3D models*, the lung extraction boosted their performance unsubstantially: from 8.27 ± 4.13 in 3*D* *non*−*extracted* models to 7.94 ± 4.13%; *p* = 0.82 in 3*D* *extracted* ones (see Table 2).

**Table 2:** Comparison of CNN-based regression models.

**Settings**	**Group 1**		**Group 2**		**p-value**
	**Models**	**Mean_1_±SD_1_**	**Models**	**Mean_2_±SD_2_**	
**Top informative view radiograms**					

B30f-B80f	CXR coronal(C)	13.49 ± 11.81	CXR sagittal(S)	10.44 ± 5.40	0.2838
**Model architectures for multiplanar assessment**					

B30f-B80f	CXR DB(C+S)	10.90 ± 6.72	CXR VR(C+S)	11.96 ± 8.30	0.9397
**Diagnostic value of multiplanar radiograms**					

B30f-B80f	CXR coronal, CXR sagittal	11.96 ± 9.23	CXR DB, CXR VR	11.43 ± 7.51	0.7016
**Effectiveness of background removal**					

B30f-B80f	CXR coronal	13.49 ± 11.81 B30f	3D-nonextracted	8.27 ± 4.13	3D-extracted	7.94 ± 4.131	0.8206
**Advantage of 3D over 2D models**					

B30f	CXR coronal, CXR sagittal	11.75 ± 8.26	3D-nonextracted	8.27 ± 4.13	0.1358
B30f	PP coronal, PP sagittal	10.66 ± 5.83	3D-extracted	7.94 ± 4.13	0.1862

#### 4.3.2. 3D vs. 2D Models

**Figure 5:**
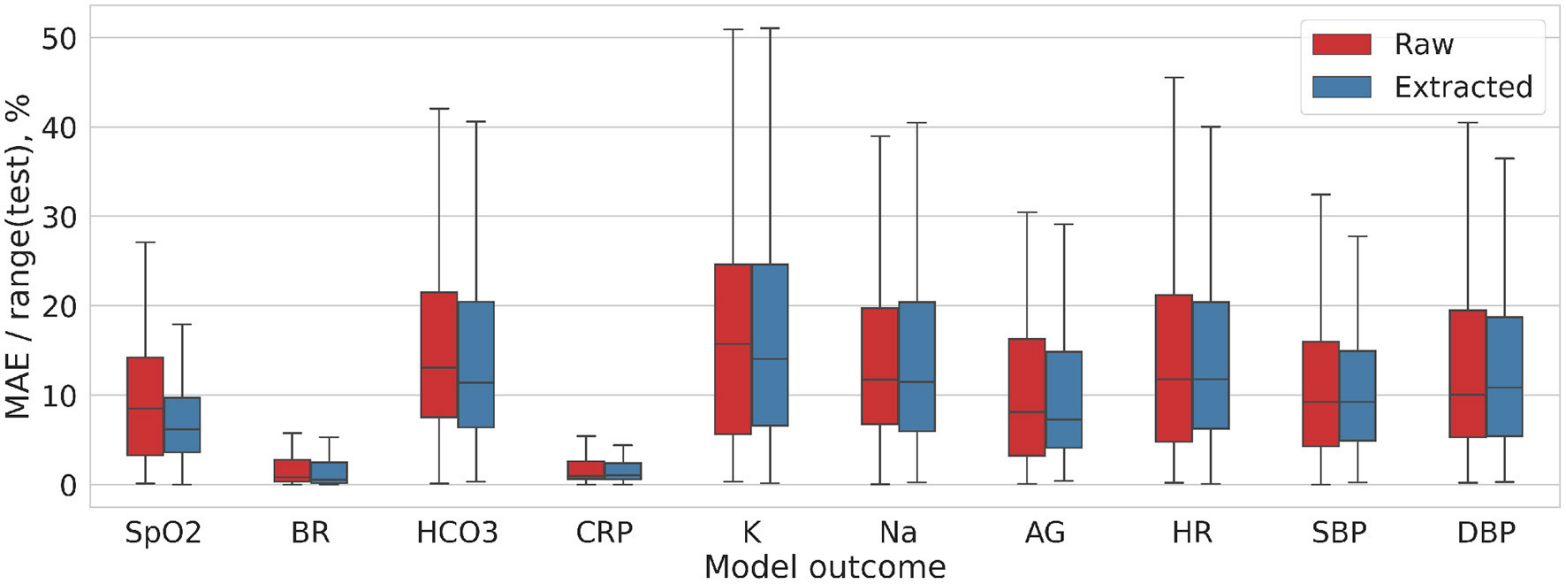
Distribution of the MAE/range(test) values for DL model trained on 3D-Nonextracted and 3D-Extracted CT images.

Figure 5 and Table 1 present the performance of the models trained on diagnostic 3D images that were reconstructed with B30f kernel. The models trained on 3D images were more accurate than those on 2D. *Before lung extraction*, the MAE/range was 11.75 ± 8.26 for algorithms trained on 2D and it reduced to 8.271 ± 4.13% with 3D data, *p* = 0.14 (all *CXR* vs. all 3*D* *non*−*extracted*). *After lung extraction*, the performance metrics were 10.66 ± 5.83 for 2D data and 7.94 ± 4.13% for 3D data, *p* = 0.18 (all *PP* vs. all 3*D* *extracted*).

### 4.4. Comparison of Reconstruction Kernels

**Figure 6:**
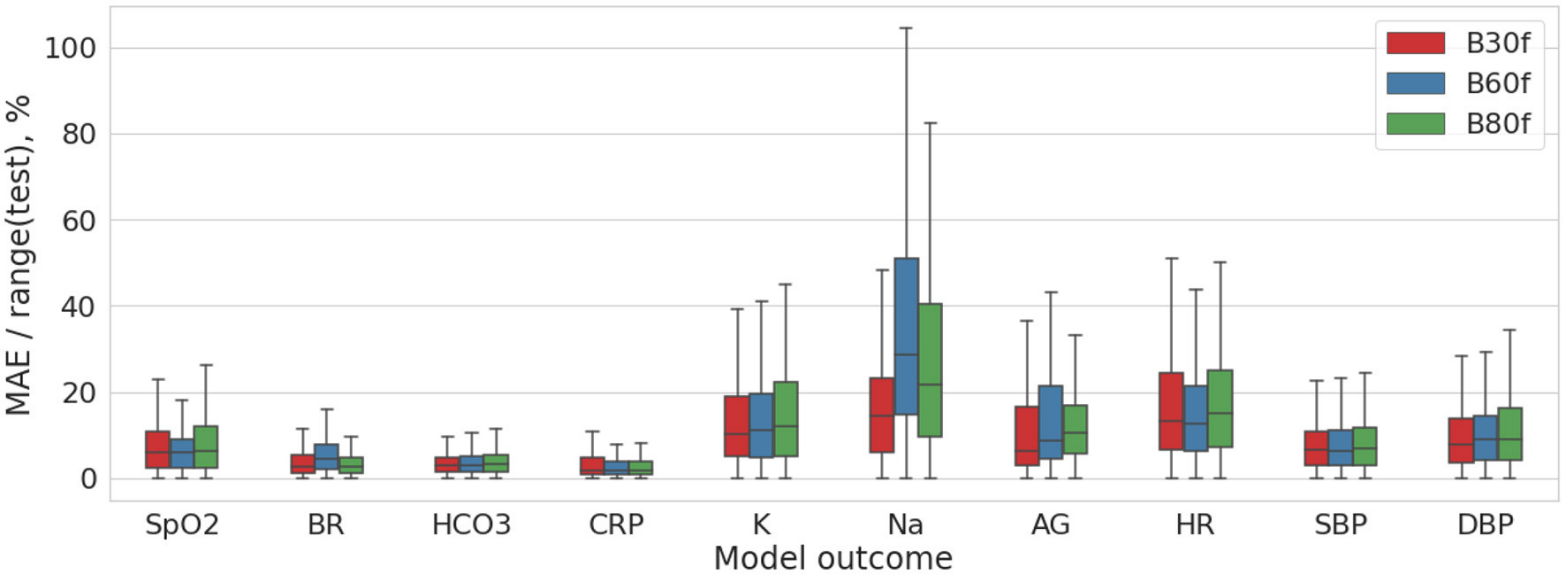
Distribution of the MAE/range(test) values for DL model trained on the multimodal 2D data (pre-processed coronal and axial CT images).

We analyzed how the settings of CT reconstruction kernels may impact the diagnostic image quality. The final subobjective of the study was to compare B30f, B60f, and B80f kernel images with regard to their potential to reflect the clinical status of the patients with coronaviral pneumonia. We trained the models predicting the level of hypoxia on images acquired with these reconstruction kernels. Notched boxplot diagrams on Figure 6 depict the high accuracy of the models especially the ones predicting the CRP level and the physiological markers of the respiratory system (breath rate, oxygen saturation, HCO_3_).

Table 1 shows insignificant variance in the accuracy of the models trained on images reconstructed with different settings. The reconstruction algorithms achieved approximately the same degree of accuracy, with the best level in B30f kernel. The top performance models were trained on a combination of the coronal and sagittal view chest radiograms and pre-processed (segmented) coronal and axial CT images (the ratio of MAE to range of values were 9.989 ± 5.614% and 10.14 ± 4.745%).

## 5. Discussion

### 5.1. Biochemical and Physiological Markers of Hypoxia Resulting From Acute Lung Injury

We observed a strong association between the structural markers of lung damage (the extent and spread of the inflammation over the respiratory tissue) and the hypoxia level. The strongest correlation was found between the oxygen saturation level and the percentage of the lung involvement each serving as the most accurate measurement of hypoxia level and COVID-19-associated lung injury correspondently. The correlation of the lung tissue damage is more remarkable with the functional parameters reflecting cardiovascular system (BR, HR) than with biochemical response (AG, *K*^+^). The reason for this is quite evident as we deal with the respiratory acidosis in COVID-19 and electrolyte imbalance commonly happens for metabolic reasons, e.g., in acute renal injury.

The data we analyzed provide a useful insight on the lung structure-function association. To realize the value of these studies, one should consider the clinical merit of the forecasted functional estimates and their limitations. For instance, “silent” (asymptomatic) hypoxia in CAP is a commonly overlooked clinical entity (34). To permit timely CAP diagnosis, physicians should employ a 6-min walk test to diagnose exertional hypoxia (35) or conduct a meticulous clinical examination (34). If considered together, the functional estimates (e.g., arterial blood gases, pulse oximetry, etc.) may provide an accurate assessment of the patient's oxygenation status and prevent poor outcomes (36, 37). In contrast to this, the structural findings of lung injury may elucidate hidden pathophysiological conditions. Some authors suggest using low-dose CT to ensure that the symptoms of hypoxia are not overlooked (11).

The association between structure and function is apparent from the reliable models that predict the hypoxia level in pneumonia patients from CT findings. The models we built show a high-accuracy prediction of the oxygen saturation and bicarbonate levels, breath and heart rates and the level of potassium and AG. A possible explanation for these findings are provided in the next few paragraphs.

#### 5.1.1. Oxygen Saturation and Hypoxemia

In our study, the prediction of the SpO_2_ level was less accurate than those of CO_2_ and breath rate. The following facts may account for this. Researchers warn treating clinicians that oxygen saturation constantly changes because of hypoxic and hypercapnic ventilatory responses, cardiac output and physical exertion. Physicians should be aware that the profound hypoxemia observed in COVID-19 may present a temporary nadir. It is incorrect to think that hypoxia alone causes tissue injury. When hypoxemia is compensated by the cardiovascular responses, it is well tolerated (17). However, when the cardiovascular system fails to compensate for the critical reduction of blood flow and oxygen delivery, acidosis develops. The cardiovascular mechanism of adjustment is an elevated cardiac output due to tachycardia with a moderate augmentation of the blood pressure. The biochemical adjustment involves an increased production of 2,3-diphosphoglycerate with cellular glycolysis and a rise in the Michaelis–Menten constant for hemoglobin.

#### 5.1.2. Hypocapnia (Hypocarbia)

The models we built predict HCO_3_ levels quite accurately with the MAE/range of 5%. This finding offers us an insight into the structure-function association. From the clinical perspective, assessment of the HCO_3_ level is mandatory for analyzing the severity of respiratory failure. Isolated monitoring of SpO_2_ is insufficient for making a decision. Hypoxia is thought to increase the respiratory rate; however, hyperventilation results in hypocapnia that may attenuate a ventilatory response to hypoxia. *In the early stage and later in mild cases* of COVID-19, hypoxemia and abnormal levels of HCO_3_ are highly unlikely to be observed. *In severe cases* of COVID-19, hypoxemia initiates a compensatory ventilatory response leading to noticeable hypocapnia (37). High carbon dioxide levels may also develop in ARDS patients but it remains unclear how hypercapnia impacts the outcome (38).

#### 5.1.3. Breath Rate

The most accurate prediction in our study was that of the breath rate as seen from the least value of MAE/range (over 4%). This means that the radiologic findings may reflect dyspnea more reliably than the other considered laboratory or physiologic parameters of hypoxia. In the mild or moderate ARDS, the subjective shortage of breath can be limited with subtle increases in the respiratory rate even if arterial hypoxemia is present. The elevated respiratory rate helps the lung to breathe out carbon dioxide, and this mitigates the sensation of dyspnea (17). Hypoxic ventilatory responses in humans vary in the number of changes in respiratory rate and tidal volume. Individual factors (e.g., age, ethnicity, obesity) may reduce the hypoxic and hypercapnic ventilatory responses thus putting these patients at a higher risk of more profound symptoms of hypoxia at clinical presentation.

#### 5.1.4. Potassium

Potassium is the primary intracellular electrolyte that produces osmotic pressure to maintain the cell volume. Normally the concentration of potassium in the serum is maintained within a relatively narrow range of 3.5–5 mEq/l (39). Hyperkalemia that we observe in a hypoxic condition is an elevation of serum potassium above 5 mEq/l. It is characteristic of systemic hypoxia which inactivates the ATPase of the sodium-potassium pump. The deficit of oxygen results in acidosis and shifts potassium from the intra- to extracellular compartment as ATP is not sufficiently replenished (40, 41).

The regression models predicting the level of potassium in our study were the least accurate ones with the MAE/range values ranging from 16 to 60%. Theoretically, the additional uptake of potassium from drugs containing the electrolyte or other sources may have confounded the study results. Contrarily, the models forecasting the level of another marker of hypoxia (AG) are quite reliable with the MAE/range values varying from 4 to 6%.

#### 5.1.5. Anion Gap

Anion Gap is the difference between measured positively (cations) and negatively (anions) charged ions with the range of normal values from +8 to +16 *mmol*/*l* (40). The gap forms because the sum of the cations excluded from Formula 1 (e.g., *Ca*^2^+) is lower than the sum of the anions ignored while calculating AG (proteins, organic acids). The AG goes up in some cases of hypoxia. *In acidosis* physicians observe either enlarged or normal values of AG (42, 43). Tissue hypoxia is present in all forms of lactic acidosis (44). A reduced AG may indicate a decrease in the albumin concentration (hypoalbuminemia) as albumin is the primary non-measured anion.

#### 5.1.6. Heart Rate

The prediction of the heart rate was inaccurate in our study: MAE/range values varied from 15 to 17%. The reason may be due to the fact that a wide variety of physiological factors act as confounders of the results of the physical examination of the patient.

### 5.2. Automatic Assessment of Two-Dimensional Diagnostic Images Acquired in a Single and Multiple Planes

Previous research has shown the reliability of the CXR for the evaluation and management of COVID-19 pneumonia (45). This finding justified the feasibility and repeatability of a human-driven quantitative CXR assessment. In their studies the severity score calculated by radiologist from 2D images showed a significant positive correlation with CRP, lactate dehydrogenase, and fever duration, as well as a negative correlation with SpO_2_. In line with these studies, we aimed to evaluate an automatic assessment of 2D diagnostic images of the chest. The regression models trained on 2D data showed a good performance.

The routine use of lateral view chest radiographs has been the subject of much debate. We tested if multiplanar CXR aids computer-driven assessment of the lung injury. The additive value turns out to be small. This corresponds to the clinical studies which showed that lateral views contribute to increased detection of pneumonia only in a small number of cases (46–49). Because of the low yield and the additional radiation exposure, researchers criticized the idea of including lateral view radiographs in epidemiological studies for trial purposes (50).

### 5.3. 3D Models of Structure-Function Association Information Noise Reduction With Lung Extraction Technique

We intended to compare the accuracy of models fed with 2D and 3D diagnostic images. According to our findings, models trained on the 3D CT images had a better performance compared to the ones fed with the 2D plane radiograms. The difference between the accuracy of the models trained on the non-extracted 2D and 3D images was approaching significance (11.75 ± 8.26 vs. 8.271 ± 4.13%; *p* = 0.14). This keeps the debate on the advance of CT over CXR in pneumonia cases opened. The indications for ordering either CT or CXR for COVID-19 patients differ among professional communities. The British Society of Thoracic Imaging recommended CT for the patients with uncertain or normal CXR findings and for the follow-up studies of the cases suspected of complications. Despite the low sensitivity (30-60%), CXR is still sufficient for some cases of COVID-19 (51) as both modalities depict similar findings (e.g, unilateral or bilateral involvement, etc.) (52). Furthermore, X-Ray machines expose patients to a lower radiation dose than CT scanners and surfaces of the machines can be more easily cleaned for infection control in such a pandemic. Because of the apparent priority to use CXR over CT, there is a lack of studies on optimal tactics of CXR assessment.

The use of 2D vs. 3D imaging in the evaluation for pneumonia has been an issue of clinical studies. One previous study showed that in 27% of cases, when both CXR and CT scans were performed, pneumonia was demonstrated on CT in case of a negative or non-diagnostic CXR (53). However, the study had a selection bias as it only included the patients whose clinical status demanded extensive imaging, i.e. physicians aimed to rule out pulmonary embolism as an explanation for the symptoms (53).

#### 5.3.1. Reduction of Information Noise With Lung Segmentation

In agreement with the findings from previous studies, we showed a slight boost in the accuracy of the models after the lung was extracted from the CT image. Lung segmentation is an important part of pulmonary image analysis. The correct detection of the organ of interest and delineation of its anatomic boundaries is crucial for the subsequent identification (29, 54) and quantification (radiomics) of diseased areas (55). Even in digital lateral chest radiographs, automated lung segmentation has been an issue of separate studies for a long time (56). This is in line with a conventional approach to pneumonia detection on CXR with a machine learning paradigm: researchers focus analysis on pixels in lungs segmented region that are contributing more toward pneumonia detection than the surrounding regions (57).

Owing to generating 2D diagnostics images from 3D, we had a unique opportunity to check if the segmentation of the lung on 2D images contributed to the accurate automatic assessment of disease severity. As seen in Table 1, the models trained on 2D diagnostic images reconstructed in coronal plane were slightly more accurate (by over 1–2%) when segmentation was applied. There are a large number of approaches to lung segmentation in computed tomography. However, the small boost in performance provided by the techniques is a reason for their limited clinical application (29). A similar but more pronounced tendency is reported for deep learning with the lung segmentation for CXR analysis of lung cancer. The pre-processed dataset without clavicle and rib bones showed a much better accuracy compared to the data without background removal (58).

### 5.4. Impact of Reconstruction Kernels

The effects of the reconstruction kernel (also referred to as algorithms or filters) on the image quality is a common issue in radiology studies (59–62). The results of these studies suggest that the selection of a kernel for an examination should be careful, and it should correspond to clinical interest. This is because image noise strongly depends on the reconstruction kernel (59). A sharper (higher resolution, edge-enhancing) kernel generates a higher spatial resolution image, but increases image noise. A smoother (lower resolution) kernel produces a more accurate representation. Both spatial resolution and image noise account for the final image quality. Technicians should utilize proper settings for image acquisition according to clinical interest (e.g., the size and appearance of the targeted structure and the general background). For example, the evaluation of small *low-contrast* structures should advance from the application of sharper high-resolution kernels (63). In contrast, the ability to detect small *high-contrast* lesions improves as the reconstruction kernel becomes smoother (64).

Regarding lung pathology, sharper image reconstruction kernels result in higher CT measurements of *emphysema* than smoothing kernels (65). In an earlier study (59), the noise obtained from B31f reconstructed images was lower than that obtained from B70f tomograms. From our data, the reconstruction kernel settings do not affect the quality of ML models. Presumably, these settings are not crucial for computer-aided diagnostics but they may still impact the visual diagnostics by radiologists.

## 6. Strengths and Limitations

The study has the following strengths and limitations. Though the symptoms of COVID-19-associated pneumonia are more pronounced compared to other viral pneumonias, the key pathophysiological mechanisms of the disease are not unique (17). Thus, the models built and the approaches used are likely to be applicable for the future outbreaks of viral pneumonias.

Another strength of the study is the practical implementation of the proposed models. There are different phenotypes of COVID-19-associated ARDS (37). Therefore, clinicians need to address whether the blood saturation values reflect the actual structural changes in the lung parenchyma. Cardiovascular compensation of hypoxia may adjust the values of oxygen saturation in the blood, thus hiding the actual lung injury.

Finally, the strength of this research is that we created regression models that provided numerical automatic assessment of lung impairment. The qualitative evaluation with classification models and categories was less accurate.

The known limitations of the study are as follows. *First*, we performed a single-center study, with all CT images acquired using a single scanner. *Second*, we worked with the CT scans acquired right on admission when the patients were hospitalized a day or two after the disease emerged and they tested positive. As the radiological findings varied across the disease phases, the models we built were not trained to work with the data typical for the intermediate and late phases of the disease. Future studies are expected to extend the utility of the ML algorithms by applying them to the results of the follow-up studies. *Third*, we tested the patients exceptionally for SARS-CoV-2. However, coinfections may have occured, and this should be considered (66).

## 7. Conclusion

- The ML algorithms trained on radiological findings can reflect morphological characteristics and pathophysiologic changes. The models reveal structure-function association. Therefore, they may contribute to a more optimal risk evaluation and disease management in COVID-19.
- Training the models on multiplane 2D images improved the performance from over 11÷12% by 0.5÷1%. The models fed with sagittal view radiograms showed higher accuracy than the models fed with coronal view, but the difference was not significant: 10.43 ± 5.40 vs. 13.49 ± 11.81%; *p* = 0.28.
- Image pre-processing with the lung segmentation technique slightly increased the accuracy of 2D models: MAE/range droped by over 1÷2%, the performance metric for non-extracted and extracted frontal view radiograms were 13.49 ± 11.81 and 11.08 ± 6.5% respectively (*p* = 0.54).
- The models trained on 3D images were more accurate than those on 2D: 8.27 ± 4.13 and 11.75 ± 8.26%, *p* = 0.14 before lung extraction; 10.66 ± 5.83 and 7.94 ± 4.13%, *p* = 0.18 after the extraction.
- The top accurate models were trained on pre-processed and segmented 3D images: MAE/range of the models predicting breath rate was 2.946%, bicarbonate level - 3.21%.
- The reconstruction kernel settings did not affect the model performance but they may have impacted visual diagnostics by radiologists.

## Data Availability Statement

The datasets presented in this study can be found in online repositories. The datasets generated for this study are available on request at the site of Big Data Analytics Center at https://bi-dac.com.

## Ethics Statement

The studies involving human participants were reviewed and approved by Department of Health Abu Dhabi (reference Number: DOH/CVDC/2020/887). Written informed consent for participation was not required for this study in accordance with the national legislation and the institutional requirements.

## Author Contributions

YS and TH contributed to the conceptual idea of the paper. YS, ML, EL, and KMD formulated the objectives. YS and EL wrote the manuscript. TH performed the statistical analysis, prepared the figures, tables for data presentation, and illustration. KMD, TT, MP, AP, PB, JG, KG, DQ, SE, AP, TL, NN, GM, NZ, TA, FZ, and JA contributed to the literature review and data analysis. All authors contributed to the article and approved the submitted version.

## Funding

This work was supported by MBRU Collaborative Research Award 31M418/21M153 (PI: KMD), UAEU CMHS research grant 12M119 (PI: KG), and Aspire grant AARE19-060 (PI: ML).

## Conflict of Interest

The authors declare that the research was conducted in the absence of any commercial or financial relationships that could be construed as a potential conflict of interest.

## Publisher's Note

All claims expressed in this article are solely those of the authors and do not necessarily represent those of their affiliated organizations, or those of the publisher, the editors and the reviewers. Any product that may be evaluated in this article, or claim that may be made by its manufacturer, is not guaranteed or endorsed by the publisher.

## Abbreviations

AG: anion gap
aPTT: activated partial thromboplastin time
ARDS: acute respiratory distress syndrome
AUC: area under the curve
BR: breath rate
CAP: community-acquired pneumonia
CI: confidence interval
COVID-19: coronavirus disease 2019
CRP: C-reactive protein
CT: computed tomography
CTS: CT score
CXR: Chest X-ray
DB: Data blending
DL: deap learning
HR: heart rate
LLL: left lower lobe
LUL: left upper lobe
MAE: mean absolute error
ML: machine learning
PaCO_2_: alveolar CO_2_ partial pressure
PaO_2_: alveolar oxygen partial pressure
PCR: polymerase chain reaction
PP: pre-processed images (extracted lungs)
PO: pulse oxymetry
PR, pathology rate: rate of lung pathology in %
RLL: right lower lobe
RML: right middle lobe
RUL: right upper lobe
SAP: SARS-CoV-2 associated pneumonia
SARS-CoV-2: severe acute respiratory syndrome-related coronavirus 2
SpO_2_: oxygen saturation measured by pulse oximetry
SaO_2_: arterial oxygen saturation
VR: voting regression.
