# Supplementary material for: Deep Learning-Based Automatic Assessment of Lung Impairment in COVID-19 Pneumonia: Predicting Markers of Hypoxia With Computer Vision
Source: Front Med (Lausanne). 2022 Jul 26;9:882190. doi: 10.3389/fmed.2022.882190 (PMC9360571; doi:10.3389/fmed.2022.882190)
Supplement: Supplementary file 1 [file Data_Sheet_1.PDF]

2D CNN

In [1]:

```
from __future__ import absolute_import, division, print_function, unicode_literals
import tensorflow as tf
from tensorflow import keras
import numpy as np
import matplotlib.pyplot as plt
import pandas as pd

from tensorflow.keras import layers
from tensorflow.keras import models
from tensorflow.keras import optimizers
from tensorflow.keras import regularizers
from tensorflow.keras.layers import BatchNormalization
from tensorflow.keras.layers import Activation
from tensorflow.keras.callbacks import EarlyStopping, CSVLogger

from sklearn.model_selection import KFold
import warnings
warnings.filterwarnings("ignore")
```

In [2]:

```
print("Num GPUs Available: ", len(tf.config.experimental.list_physical_devices('GPU'
# tf.debugging.set_log_device_placement(True)
```

```
Num GPUs Available:  3
```

In [3]:

```
protocol = 'B30f'
cols = ['lab_Potassium_Lvl','Heart_rate','Breath_rate','Systolic_blood_pressure','Di
original_dataset_dir  = "../CT_DBs/" + protocol + "/Averaged_resized/"
dataset_dir = "../CT_DBs/" + protocol + "/DL_datasets/"
col = cols[0]
```

In [4]:

```
# load the dataset
dataset = np.load(dataset_dir + col + ".npy")
labels = pd.DataFrame()
labels[col] = np.loadtxt(dataset_dir + col + '.csv',dtype=float)
```

In [5]:

```
dataset.shape
```

Out[5]:

```
(625, 150, 250, 3)
```

Preprocess the data

The data must be preprocessed before training the network. The pixel values fall in the range of 0 to 255 in every image.

In [7]:

```
plt.figure()
plt.imshow(dataset[1],  cmap=plt.cm.binary)
plt.grid(False)
plt.show()
```

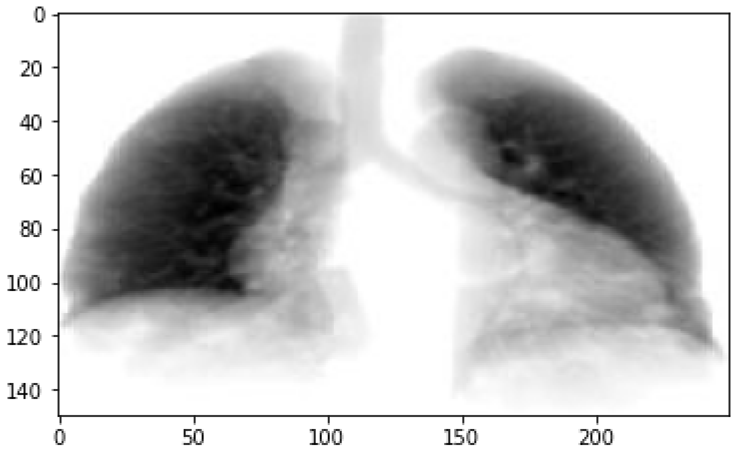

In [6]:

```
dataset = dataset/255.
```

To verify that the data is in the correct format, we may display the first 50 images from the *training set* and display the class name below each image.

In [10]:

```
def plot_grid(images,labels):
    plt.figure(figsize=(10,10))
    for i in range(25):
        plt.subplot(5,5,i+1)
        plt.xticks([])
        plt.yticks([])
        plt.grid(False)
        plt.imshow(images[i], cmap=plt.cm.binary)
        plt.xlabel(labels[col][i])
    plt.show()
```

In [11]:

```
plot_grid(dataset,labels)
```

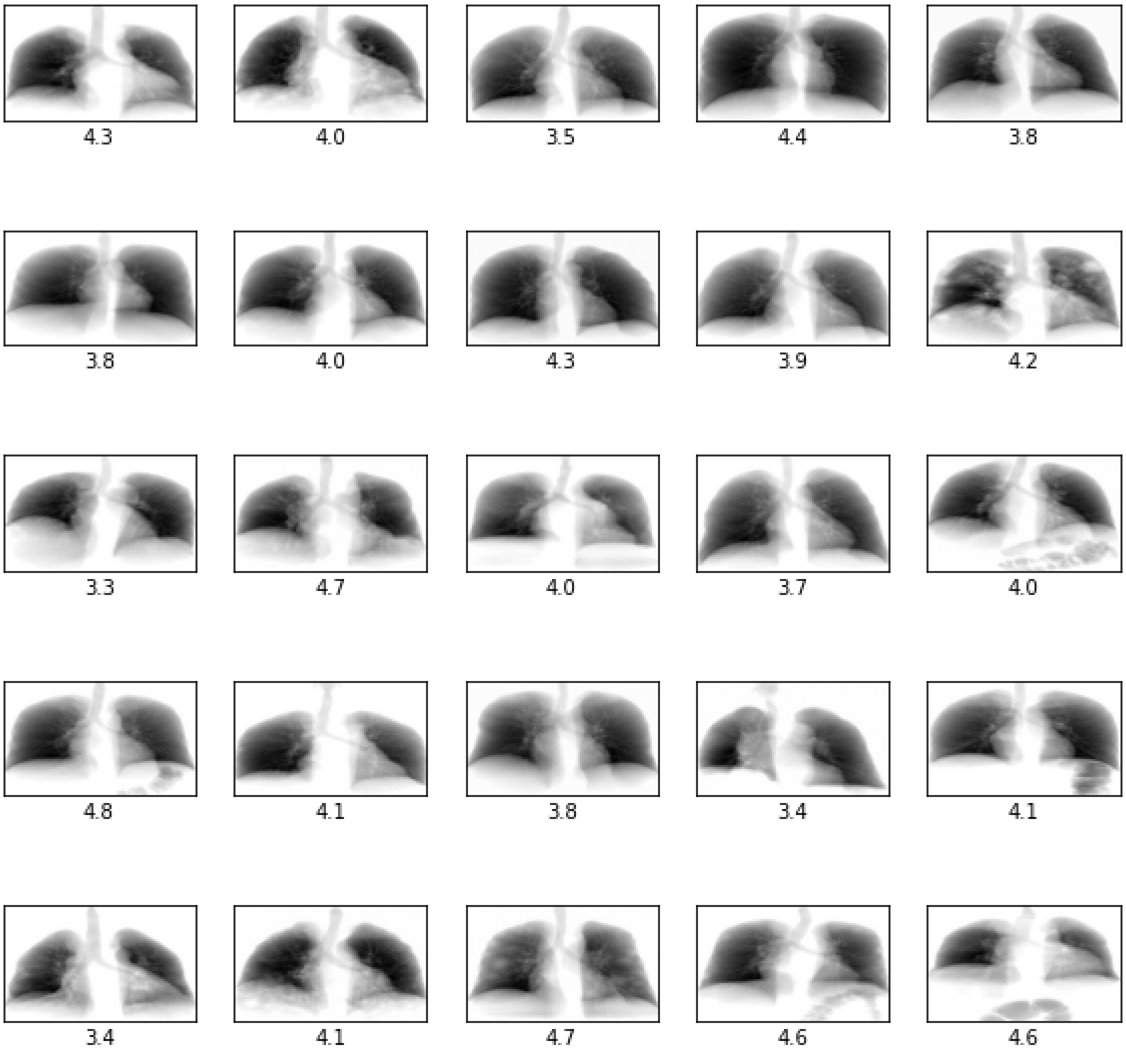

Model

In [7]:

```
img_rows  = 150
img_cols = 250
img_channels = 3
```

Design

In [4]:

```
# from keras import applications
def build_model_res():
    model = models.Sequential()
#     resnext.ResNeXt50
    base_model = keras.applications.resnet50.ResNet50(weights= None, include_top=Fal
    model.add(base_model)
    model.add(layers.Flatten())
#     model.add(layers.Dense(256, activation='relu'))
    model.add(layers.Dense(64, activation='relu'))
    model.add(layers.Dense(32, activation='relu'))
    model.add(layers.Dense(1))
    from tensorflow.keras import optimizers
    model.compile(loss='mean_squared_error',
        optimizer=optimizers.RMSprop(learning_rate=0.001, rho=0.9),
        metrics=['mean_absolute_error', 'mean_squared_error'])
    return model
# model1 = build_model_res()

# model1.summary()
```

In [5]:

```
def build_model():
    model = models.Sequential()
    model.add(layers.Conv2D(64, (3, 3),activation='relu',kernel_regularizer=regulari
    model.add(layers.MaxPooling2D((2, 2)))
    model.add(BatchNormalization())
    model.add(layers.Conv2D(64, (3, 3), kernel_regularizer=regularizers.l2(0.001), a
    model.add(layers.MaxPooling2D((2, 2)))
    model.add(layers.Conv2D(64, (3, 3), kernel_regularizer=regularizers.l2(0.001), a
    model.add(layers.MaxPooling2D((2, 2)))
    model.add(layers.Conv2D(32, (3, 3), kernel_regularizer=regularizers.l2(0.001), a
    model.add(layers.MaxPooling2D((2, 2)))
    model.add(layers.Conv2D(32, (3, 3), kernel_regularizer=regularizers.l2(0.001), a
    model.add(layers.MaxPooling2D((2, 2)))
    model.add(layers.Flatten())
    model.add(layers.Dense(64, activation='relu'))
    model.add(layers.Dense(32, activation='relu'))
    model.add(layers.Dense(1))
    from tensorflow.keras import optimizers
    model.compile(loss='mean_squared_error',
                optimizer=optimizers.RMSprop(learning_rate=0.001, rho=0.9),
                metrics=['mean_absolute_error', 'mean_squared_error'])


    return model
```

In [13]:

```
def train_evaluate(model, dataset, labels, to_predict):
    num_folds = 5
    kfold = KFold(n_splits=num_folds, shuffle = False)
    res = pd.DataFrame()
    EPOCHS = 150
    n_train = len(dataset)
    batch_size = 32
    callbacks = [keras.callbacks.EarlyStopping(
                    monitor='val_loss',
                    patience=20,),
                keras.callbacks.ReduceLROnPlateau(monitor='val_loss', factor = 0.2,
                    patience=10, min_lr=0.000001) ]
    for train, test in kfold.split(dataset, labels):
        targets = labels[to_predict].values
        model = build_model()
        model.fit(dataset[train], targets[train], epochs=EPOCHS, validation_split =
        loss, mae, mse = model.evaluate(dataset[test], targets[test], verbose=0)
#          print("Testing set Mean Abs Error: {:5.2f}".format(mae))
        test_predictions = model.predict(dataset[test]).flatten()
        test_ = pd.DataFrame()
        test_[to_predict] = targets[test]
        test_['pred'] = test_predictions
        test_['error'] = test_[to_predict] - test_.pred
        test_['abs_error'] = abs(test_[to_predict] - test_.pred)
        test_['test'] = to_predict
        res = pd.concat([res, test_])
    return res
```

In [19]:

```
def test_res(res, col):
    res_ = pd.DataFrame()
    res_['to_predict'] = res[col].mean()
    res_['pred'] = res['pred'].mean()
    res_['error'] = res['error'].mean()
    res_['MAE'] = res['abs_error'].mean()
    res_['MAE/range, %'] = round(res_['MAE']* 100/(res.describe()[col][7]-res.descri
    return res_
```

In [20]:

```
def train_test(protocol):
    cols = ['lab_Potassium_Lvl','Heart_rate','Breath_rate','Systolic_blood_pressure'
    dataset_dir = "../CT_DBs/" + protocol + "/DL_datasets/"
    res = pd.DataFrame()
    for col in cols:
        # load the dataset
        dataset = np.load(dataset_dir + col + ".npy")
        labels = pd.DataFrame()
        labels[col] = np.loadtxt(dataset_dir + col + '.csv',dtype=float)
        dataset = dataset/255.
        model = build_model()
        tmp = train_evaluate(model, dataset, labels, col)
        results = test_res(tmp, col)
        res = pd.concat([res, results])
    return res
```

Training and performance evaluation

In [ ]:

```
train_test("B30f")
```
