# Supplementary material for: Deep Learning-Based Automatic Assessment of Lung Impairment in COVID-19 Pneumonia: Predicting Markers of Hypoxia With Computer Vision
Source: Front Med (Lausanne). 2022 Jul 26;9:882190. doi: 10.3389/fmed.2022.882190 (PMC9360571; doi:10.3389/fmed.2022.882190)
Supplement: Supplementary file 2 [file Data_Sheet_2.PDF]

3D Image Regression from CT Scans

In [2]:

```
import os
import zipfile
import numpy as np
import tensorflow as tf
import pandas as pd
from PIL import Image
from tensorflow import keras
from tensorflow.keras import layers
from sklearn.model_selection import train_test_split
```

Let's read the paths of the CT scans from the class directories.

In [3]:

```
protocol = "B30f"
col = 'Breath_rate'
dataset_dir = "../CT_DBs/" + protocol + "/3D_DL_Datasets/Extracted/"
dataset = np.load(dataset_dir + col + "_3D.npy")
labels = np.loadtxt(dataset_dir + col + '_3D.csv',dtype=float)
X, X_test, y, y_test = train_test_split(dataset, labels, test_size=0.20, random_stat
X_train, X_val, y_train, y_val = train_test_split(X, y, test_size=0.20, random_state
print(X_train.shape, X_val.shape, X_test.shape, y_train.shape, y_val.shape, y_test.s
```

```
(456, 128, 128, 64) (114, 128, 128, 64) (143, 128, 128, 64) (456,) (11
4,) (143,)
```

Data augmentation

In [4]:

```
import random
from scipy import ndimage
@tf.function
def rotate(volume):
    """Rotate the volume by a few degrees"""

    def scipy_rotate(volume):
        # define some rotation angles
        angles = [-25,-20, -10, -5, 5, 10, 20,25]
        # pick angles at random
        angle = random.choice(angles)
        # rotate volume
        volume = ndimage.rotate(volume, angle, reshape=False)
        volume[volume < 0] = 0
        volume[volume > 1] = 1
        return volume

    augmented_volume = tf.numpy_function(scipy_rotate, [volume], tf.float32)
    return augmented_volume
```

In [ ]:

```
def train_preprocessing(volume, label):
    # Rotate volume
    volume = rotate(volume)
    volume = tf.expand_dims(volume, axis=3)
    return volume, label

def validation_preprocessing(volume, label):
    volume = tf.expand_dims(volume, axis=3)
    return volume, label
```

While defining the train and validation data loader, the training data is passed through and augmentation function which randomly rotates volume at different angles. Note that both training and validation data are already rescaled to have values between 0 and 1.

In [5]:

```
# Define data loaders.
train_loader = tf.data.Dataset.from_tensor_slices((X_train, y_train))
validation_loader = tf.data.Dataset.from_tensor_slices((X_val, y_val))

batch_size = 2
# Augment the on the fly during training.
train_dataset = (
    train_loader.shuffle(len(X_train))
    .map(train_preprocessing)
    .batch(batch_size)
    .prefetch(2)
)
# Only rescale.
validation_dataset = (
    validation_loader.shuffle(len(X_val))
    .map(validation_preprocessing)
    .batch(batch_size)
    .prefetch(2)
)
```

Visualize an augmented CT scan.

In [6]:

```
import matplotlib.pyplot as plt

data = train_dataset.take(1)
images, labels = list(data)[0]
images = images.numpy()
image = images[0]
print("Dimension of the CT scan is:", image.shape)
plt.imshow(np.squeeze(image[:, :, 30]), cmap="gray")
```

```
Dimension of the CT scan is: (128, 128, 64, 1)
```

Out[6]:

```
<matplotlib.image.AxesImage at 0x7fbff405c668>
```

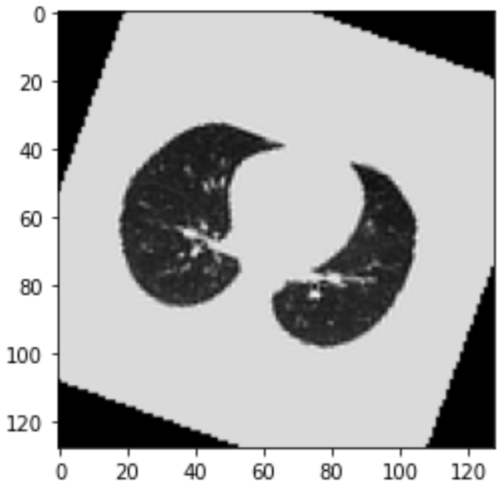

Since a CT scan has many slices, let's visualize a montage of the slices.

In [7]:

```
def plot_slices(num_rows, num_columns, width, height, data):
    """Plot a montage of 20 CT slices"""
    data = np.rot90(np.array(data))
    data = np.transpose(data)
    data = np.reshape(data, (num_rows, num_columns, width, height))
    rows_data, columns_data = data.shape[0], data.shape[1]
    heights = [slc[0].shape[0] for slc in data]
    widths = [slc.shape[1] for slc in data[0]]
    fig_width = 12.0
    fig_height = fig_width * sum(heights) / sum(widths)
    f, axarr = plt.subplots(
        rows_data,
        columns_data,
        figsize=(fig_width, fig_height),
        gridspec_kw={"height_ratios": heights},
    )
    for i in range(rows_data):
        for j in range(columns_data):
            axarr[i, j].imshow(data[i][j], cmap="gray")
            axarr[i, j].axis("off")
    plt.subplots_adjust(wspace=0, hspace=0, left=0, right=1, bottom=0, top=1)
    plt.show()


# Visualize montage of slices.
# 4 rows and 10 columns for 100 slices of the CT scan.
plot_slices(4, 10, 128, 128, image[:, :, 20:60])
```

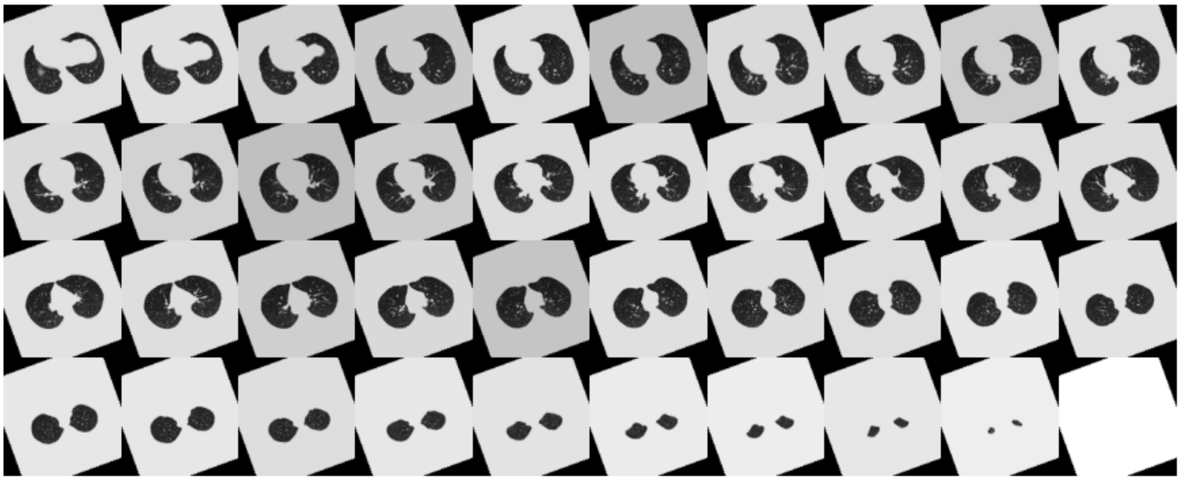

Define a 3D convolutional neural network

To make the model easier to understand, we structure it into blocks. The architecture of the 3D CNN used in this example is based on [this paper (https://arxiv.org/abs/2007.13224)](https://arxiv.org/abs/2007.13224).

In [8]:

```
def get_model(width=128, height=128, depth=64):
    inputs = keras.Input((width, height, depth, 1))

    x = layers.Conv3D(filters=64, kernel_size=3, activation="relu")(inputs)
    x = layers.MaxPool3D(pool_size=2)(x)
    x = layers.Conv3D(filters=128, kernel_size=3, activation="relu")(x)
    x = layers.MaxPool3D(pool_size=2)(x)
    x = layers.Conv3D(filters=256, kernel_size=3, activation="relu")(x)
    x = layers.MaxPool3D(pool_size=2)(x)
    x = layers.GlobalAveragePooling3D()(x)
    x = layers.Dense(units=512, activation="relu")(x)
    x = layers.Dropout(0.3)(x)

    outputs = layers.Dense(units=1)(x)

    # Define the model.
    model = keras.Model(inputs, outputs, name="3D-CNN")
    initial_learning_rate = 0.0001
    lr_schedule = keras.optimizers.schedules.ExponentialDecay(
        initial_learning_rate, decay_steps=100000, decay_rate=0.96, staircase=True
    )
    model.compile(
    loss='mean_absolute_error',
    optimizer=keras.optimizers.Adam(learning_rate=0.0001, beta_1=0.9, beta_2=0.999,
    metrics=['mean_absolute_error', 'mean_squared_error']
)

    return model

# Build and compile model
model = get_model(width=128, height=128, depth=64)
model.summary()
```

```
Model: "3D-CNN"
_________________________________________________________________
Layer (type)                 Output Shape              Param #
=================================================================
input_1 (InputLayer)         [(None, 128, 128, 64, 1)] 0
_________________________________________________________________
conv3d (Conv3D)              (None, 126, 126, 62, 64)  1792
_________________________________________________________________
max_pooling3d (MaxPooling3D) (None, 63, 63, 31, 64)    0
_________________________________________________________________
conv3d_1 (Conv3D)            (None, 61, 61, 29, 128)   221312
_________________________________________________________________
max_pooling3d_1 (MaxPooling3 (None, 30, 30, 14, 128)   0
_________________________________________________________________
conv3d_2 (Conv3D)            (None, 28, 28, 12, 256)   884992
_________________________________________________________________
max_pooling3d_2 (MaxPooling3 (None, 14, 14, 6, 256)    0
_________________________________________________________________
global_average_pooling3d (Gl (None, 256)               0
_________________________________________________________________
dense (Dense)                (None, 512)               131584
_________________________________________________________________
dropout (Dropout)            (None, 512)               0
_________________________________________________________________
dense_1 (Dense)              (None, 1)                 513
=================================================================
```

```
Total params: 1,240,193
Trainable params: 1,240,193
Non-trainable params: 0
_________________________________________________________________
```

Train model

In [9]:

```
def train_evaluate_split(protocol,mode, train_dataset, validation_dataset, X_test, y
    res = pd.DataFrame()
    EPOCHS = 100
    # Split data in the ratio 70-30 for training and validation.
    batch_size = 2
    checkpoint_cb = keras.callbacks.ModelCheckpoint( "CSV/R231CovidModel/" + protoco
    early_stopping_cb = keras.callbacks.EarlyStopping(monitor="val_loss", patience=2
    reduce_lr_plateu_cb =  keras.callbacks.ReduceLROnPlateau(monitor='val_loss', fac
    callbacks = [checkpoint_cb, early_stopping_cb, reduce_lr_plateu_cb]
    with tf.device("gpu:0"):
        model = get_model()
        model.fit( train_dataset,
                   validation_data=validation_dataset,
                   shuffle = True,
                   epochs=EPOCHS,
                   verbose=2,
                   callbacks=callbacks
        )
        loss, mae, mse = model.evaluate(X_test, y_test, verbose=0)
        print("Testing set Mean Abs Error: {:5.2f}".format(mae))
        test_predictions = model.predict(X_test).flatten()
        test_ = pd.DataFrame()
        test_[to_predict] = y_test
        test_['pred'] = test_predictions
        test_['error'] = test_[to_predict] - test_.pred
        test_['abs_error'] = abs(test_[to_predict] - test_.pred)
        test_['test'] = to_predict
        res = pd.concat([res, test_])
        return res
```

In [10]:

```
cols = ['SpO2','Breath_rate','lab_CO2', 'lab_C_Reactive_Prot',  'lab_Potassium_Lvl',
       'Heart_rate','Systolic_blood_pressure','Diastolic_blood_pressure']
#     train the model and evaluate its performance
def train_test(protocol,mode,cols):
    dataset_dir = "../CT_DBs/" + protocol + "/3D_DL_Datasets/" + mode + "/"
    with tf.device("gpu:0"):
        res = pd.DataFrame()
        for col in cols:
            # load the dataset
            dataset = np.load(dataset_dir + col + "_3D.npy")
#                 labels = pd.DataFrame()
            labels = np.loadtxt(dataset_dir + col + '_3D.csv',dtype=float)
            model = get_model()
            X, X_test, y, y_test = train_test_split(dataset, labels, test_size=0.20,
            X_train, X_val, y_train, y_val = train_test_split(X, y, test_size=0.20,
            # Define data loaders.
            train_loader = tf.data.Dataset.from_tensor_slices((X_train, y_train))
            validation_loader = tf.data.Dataset.from_tensor_slices((X_val, y_val))
            batch_size = 8
            # Augment the on the fly during training.
            train_dataset = (
                train_loader.shuffle(len(X_train))
                .map(train_preprocessing)
                .batch(batch_size)
                .prefetch(2)
            )
            # Only rescale.
            validation_dataset = (
                validation_loader.shuffle(len(X_val))
                .map(validation_preprocessing)
                .batch(batch_size)
                .prefetch(2)
            )
            tmp = train_evaluate_split(protocol, mode, train_dataset, validation_dat
            tmp.to_csv("CSV/R231CovidModel/" + protocol + "/3D/" + mode + '/' + "_pr
            results = test_res(tmp, col)
            res = pd.concat([res, results])
        res.to_csv("CSV/R231CovidModel/" + protocol + "/3D/" + mode + '/' + "report.
        print(res)
        return res
```

In [11]:

```
# performance evaluation
def test_res(res, col):
    res_ = pd.DataFrame()
    res_['actual'] = [res[col].mean()]
    res_['pred'] = [res['pred'].mean()]
    res_['error'] = [res['error'].mean()]
    res_['MAE'] = [res['abs_error'].mean()]
    res_['MAE/range in %'] = [round(res_['MAE'][0] * 100/(res.describe()[col][7]-res
    res_['test'] = col
    return res_
```

In [1]:

```
train_test('B30f','Extracted',cols)
```
